# “Whilst you are here…” Acceptability of providing advice about screening and early detection of other cancers as part of the breast cancer screening programme

**DOI:** 10.1111/hex.13330

**Published:** 2021-08-08

**Authors:** Suzanne E. Scott, Betul Rauf, Jo Waller

**Affiliations:** ^1^ Centre for Oral, Clinical and Translational Sciences, Faculty of Dentistry, Oral and Craniofacial Sciences King's College London London UK; ^2^ School of Cancer & Pharmaceutical Sciences, Cancer Prevention Group King's College London London UK

**Keywords:** acceptability, early diagnosis, intervention, teachable moment

## Abstract

**Objectives:**

This research aimed to assess women's willingness to receive advice about cervical and bowel cancer screening participation and advice on cancer symptom awareness when attending breast cancer screening.

**Methods:**

Women (*n* = 322) aged 60–64 years, living in the United Kingdom, who had previously taken part in breast cancer screening were recruited via a market research panel. They completed an online survey assessing willingness to receive advice, the potential impact of advice on breast screening participation, prospective acceptability and preferences for mode and timing of advice.

**Results:**

Most women would be willing to receive information about cervical (86%) and bowel cancer screening (90%) and early symptoms of other cancers (92%) at a breast cancer screening appointment. Those who were not up to date with cervical cancer screening were less willing. Prospective acceptability was high for all three forms of advice and was associated with willingness to receive advice. Women would prefer to receive advice through a leaflet (41%) or discussion with the mammographer (30%) either before the appointment (27%), at the appointment (44%) or with their results (22%).

**Conclusions:**

While there is high willingness and high acceptability towards using breast cancer screening as a teachable moment for advice about prevention and early detection of other cancers, some women find it unacceptable and this may reduce their likelihood of attending a breast screening appointment.

**Patient or Public Contribution:**

This study focused on gaining women's insights into potential future initiatives to encourage screening and early diagnosis of cancer. Members of the public were also involved in piloting the questionnaire.

## 1 INTRODUCTION

Diagnosis of cancer at an early stage is one of the main determinants of prognosis. In England, when breast cancer is detected at an early stage, the 5‐year survival rate approaches 98%. In contrast, women who are diagnosed with advanced‐stage breast cancer have a 26% five‐year survival rate.^1^, ^2^ For cervical and bowel cancer, the 5‐year survival rate for early‐stage disease is also over 90%, but reduces to below 20% for those with advanced‐stage disease.^2^, ^3^, ^4^, ^5^ It is therefore imperative to detect cancer early for more successful outcomes.

One means of prevention and early detection of cancer is participation in screening programmes to detect asymptomatic cancer or precancer. Another is raising awareness and encouraging timely help‐seeking for those with symptoms. In England, the majority of cancers are diagnosed via presentation with symptoms (80%) rather than through screening (6%).^6^ Therefore, ways to promote both screening uptake and timely symptom presentation are required.

In the United Kingdom, screening programmes exist for breast, cervical and bowel cancer. For breast cancer, women aged 50–70 years are invited to undergo an X‐ray of the breasts (mammogram) every 3 years. Cervical cancer screening is targeted at women aged 25–64 years. Women are initially invited every 3 years, and then every 5 years from age 50. Cervical screening originally involved a Pap smear with cytology testing. More recently, this has moved to initial human papillomavirus (HPV) testing with cytology triage for those who are HPV positive. Bowel cancer screening is offered to all adults aged 60–74 years (50–74 in Scotland) and requires a stool sample every 2 years (initially this involved guaiac faecal occult blood testing, but now uses a faecal immunochemical test). Previously, flexible sigmoidoscopy (‘bowel scope’) screening was offered at age 55 years, but this is no longer included in the NHS bowel cancer screening programme. The uptake of cancer screening is variable, with the lowest uptake for bowel cancer screening. Although it is higher for breast and cervical screening, coverage has been decreasing over time.^7^, ^8^, ^9^

A recent study investigated how many women aged 60–65 years (the only group eligible for all three cancer screening programmes in England) participated in all three during their last invitation round.^10^ The study revealed that although 90% participated in at least one screening programme, only 35% attended all three. Thirty‐seven percent of women attended two of the screening programmes and seventeen percent attended one screening programme. Ten percent participated in none of the programmes.

One way to increase participation in cancer screening is through reminders such as personalized phone calls, letters and text messages.^7^, ^11^, ^12^ A further way of encouraging screening uptake could be advice to take part in one type of cancer screening when attending another. In this way, attendance at cancer screening could act as a ‘teachable moment’ for behaviour change, affirming the current choice to attend the screening to motivate engagement with other screening programmes. Teachable moments represent a time in which people are more likely to examine how their habits can impact their physical health.^13^ They have revealed favourable results, including increased knowledge and safer behaviours.^14^ However, it is not known whether women would be willing to receive advice about screening and early detection of other cancers when attending for cancer screening and whether they would find that acceptable. Yet this is key: For an intervention to be as successful and effective as possible, it is necessary to ensure acceptability to both the people who deliver the intervention and those who receive it.^15^, ^16^ Recent developments in the study of acceptability have advocated that acceptability is a multi‐faceted construct reflecting anticipated or experienced cognitive and emotional responses to an intervention. The Theoretical Framework of Acceptability (TFA)^15^, ^16^ outlines seven component constructs such as burden, perceived effectiveness and ethicality that may contribute to perceptions of prospective, concurrent or retrospective acceptability. Yet, at present, measurement of these constructs and comparison of acceptability across different interventions remains challenging due to the lack of validated measures.

There is some existing research on willingness to receive lifestyle advice within screening programmes. The results revealed that the majority of participants expressed willingness to receive lifestyle advice at cervical, breast, lung and bowel cancer screenings. However, a smaller number indicated that they would be less likely to take part in lung cancer screening if lifestyle advice was provided.^17^, ^18^ More detailed information on acceptability is needed and views on advice about screening and early detection of other cancers have not been investigated. Just as barriers and uptake differ between screening programmes,^19^ acceptability may also differ with regard to different types of advice.

This study focuses on breast cancer screening as a teachable moment to encourage screening and early detection of other cancers. We aimed to investigate and compare women's willingness to receive advice about cervical cancer screening participation, bowel cancer screening participation and advice on cancer symptom awareness when attending breast cancer screening and women's prospective acceptability of receiving such advice. A secondary objective was to develop a measure of acceptability underpinned by the TFA, to support these investigations.

## 2 METHODS

### 2.1 Study design

The study involved a cross‐sectional online survey of women eligible for all three national screening programmes.

### 2.2 Participants

Women fulfilled the inclusion criteria if they were aged between 60 and 64 years, lived in the United Kingdom and had previously taken part in the UK national breast cancer screening programme.

### 2.3 Procedure

A market research agency with a large panel of members, Dynata (www.Dynata.com), was used to recruit participants to the online survey in June 2020. The agency circulated the study link to women in the United Kingdom aged 60–64 years. Through Office 365 Microsoft Forms, women were presented with an online information sheet explaining the purpose of the study and were asked to complete a consent form before starting the questionnaire (see below and File S1) if they wished to take part and fulfilled the inclusion criteria. Upon completion of the questionnaire, participants were thanked for their time and redirected back to the Dynata website to qualify for a Dynata‐administered incentive. Completion of each questionnaire item was compulsory to progress to the next item, with the exception of postcode and history of cancer. Ethical approval was granted by King's College London Ethics Committee (Ref: MRSU‐19/20‐18876).

### 2.4 Measures

#### 2.4.1 Sociodemographics variables

Participants were asked to confirm their age, educational attainment, marital status, ethnicity and postcode. Postcode was subsequently converted into indices of multiple deprivation deciles,^20^ with 1 representing the most deprived decile and 10 the least deprived decile.

#### 2.4.2 Health and health‐related behaviours

Participants were asked to report their recent daily fruit and vegetable consumption, weekly physical activity, smoking status and history of cancer. For analyses, those who consumed five or more portions per day were compared to those who ate fewer fruit and vegetables.

#### 2.4.3 Screening history

Participants were asked to report the timing of their last mammography, cervical screening attendance and participation in bowel cancer screening (either home testing kits or bowel scope screening).

#### 2.4.4 Willingness to receive advice

Willingness to receive advice about early detection of other cancers was measured using three versions of the item developed by Stevens et al.^18^: ‘Would you be willing to receive advice about cervical cancer screening [bowel cancer screening/early symptoms of different types of cancer] when you are at a breast screening appointment?’. Five response options ranged from ‘Yes, definitely’ to ‘No, definitely not’. Responses were dichotomized as willing (*Yes, definitely; Yes, probably*) and not willing (*No, probably not; No, definitely not; Not sure*) for the analysis.

#### 2.4.5 Impact of advice about early detection of other cancer on breast screening participation

Participants were asked three versions of the following item: *‘*If you knew you would receive advice about cervical cancer screening [bowel cancer screening/early symptoms of different types of cancer] as part of a breast cancer screening appointment, would this make you less likely to attend breast screening?’ This is adapted from the measure used by Stevens et al.^18^ Five response options were offered and dichotomized for analysis (‘No likely adverse impact on attendance’ [*No, definitely not; No probably not*] and ‘Possible adverse impact on attendance’ [*Yes, probably; Yes, definitely; Not sure*]).

#### 2.4.6 Prospective acceptability

Acceptability of receiving advice on cervical screening, bowel screening and cancer symptoms was measured using two subscales reflecting cognitive acceptability and affective acceptability. Every item was asked in relation to each type of advice. This was a newly developed measure for this study. Items were developed to capture the domains of the TFA^16^ (e.g., affective attitude, burden, perceived effectiveness, ethicality, intervention coherence and opportunity costs). Draft items were piloted with 10 women to ensure readability and ease of completion. Psychometric evaluation of the scale is reported in File S2.

#### 2.4.7 Cognitive acceptability

Eight items assessed cognitive acceptability, reflecting the TFA^16^ domains of perceived burden, coherence, opportunity costs and efficacy of the intervention. Higher scores on this subscale (range: 8–40) indicate a higher level of acceptability. This subscale demonstrated good internal reliability in relation to receiving advice about cervical cancer screening (Cronbach's *α* = .88), bowel cancer screening (Cronbach's *α* = .90) and symptoms of cancer (Cronbach's *α* = .92).

#### 2.4.8 Affective acceptability

Five reverse‐scored items assessed affective acceptability reflecting the perceived emotional consequence of the intervention. Higher scores on this subscale (range: 5–25) indicate lower levels of negative emotions and, as such, a higher level of affective acceptability. This subscale demonstrated good internal reliability in relation to receiving advice about cervical cancer screening (Cronbach's *α* = .92), bowel cancer screening (Cronbach's *α* = .93) and symptoms of cancer (Cronbach's *α* = .92).

#### 2.4.9 Preferred mode and timing of advice

Participants were asked two general questions on their preferred mode and timing of advice about screening and early detection of other cancers. For mode, women were asked to select one of the following as their preferred way of receiving advice: video; leaflet; website; app; discussion with mammographer; discussion with GP; or discussion with practice nurse. For timing, as in the study by Stevens et al.,^18^ women were asked when they would prefer to receive advice on early detection of other cancers, with response options including ‘Before I attend the breast screening appointment’, ‘At the breast screening appointment’, ‘With my breast screening results (around two weeks after attending screening)’, ‘2–4 weeks after attending breast screening’, ‘1–3 months after attending breast screening’, ‘More than 3 months after attending breast screening’ and ‘Not at all’.

### 2.5 Analyses

Two quality assurance measures for online surveys were used before data analysis. First, ‘speeders’, participants who completed the online survey in less than a quarter of the average survey time, were excluded from analyses. Second, participants who did not respond correctly to a direct question (*‘*To make sure you're reading the questions carefully, we'd like you to select the “Agree” response to this item’) were also excluded.

Using previous estimates of willingness to receive lifestyle advice at breast cancer screening,^17^ sample size calculations indicated that the study would require a minimum of 255 participants to estimate willingness to receive advice about screening and early detection of cancer with 5% precision and 95% confidence. Descriptive analyses explored willingness to receive advice about early detection of other cancers as part of the breast cancer screening programme, the potential impact of advice on screening uptake, acceptability of receiving advice and preferences as to the mode/timing of receiving advice. Cochran's *Q* and McNemar's tests were used to investigate the differences between advice about cervical, bowel and early symptoms of different types of cancer. Friedman's and Wilcoxon signed‐rank tests were used to determine differences in the acceptability between the three types of advice. Logistic regression models were conducted to explore factors associated with willingness to receive advice with acceptability, sociodemographics and screening history as the independent variables. Variables associated with willingness to receive advice at the univariate level were entered into multivariable logistic regression (direct entry). Where significance testing was necessary for the interpretation of results, *p*< .05 was used.

## 3 RESULTS

*N *= 367 women fulfilled the inclusion criteria and completed the questionnaire. Forty‐five participants were removed from analysis following the quality assurance checks (*n *= 23 ‘speeders’; *n* = 22 answered the quality check question incorrectly), resulting in a sample of *n* = 322. Table 1 reports the sample characteristics. The majority of participants were married (*n* = 208, 65%), of White ethnicity (*n *= 309, 96%) and educated to below bachelor's degree level (*n *= 196, 61%). A substantial proportion were current or ex‐smokers (*n* = 129, 40%). The majority of women did not meet the recommended guidelines for fruit/vegetable consumption (*n* = 215, 67%). A total of 24% (*n* = 78) participants indicated that they did no physical exercise at all. The majority of participants were up to date with breast cancer screening (*n* = 271, 84%); somewhat fewer were up to date with cervical screening (*n* = 217, 67%) and bowel screening (*n* = 254, 79%).

**Table 1:** Participant characteristics

Variable			*N*	%
Sociodemographics	Age (years)	60	58	18.0
		61	59	18.3
		62	68	21.1
		63	70	21.7
		64	67	20.8
	Marital status	Married or civil partner	208	64.6
		Separated or divorced	49	15.2
		Single	41	12.7
		Widowed	24	7.5
	Ethnicity	Black, Asian and Minority Ethnic	13	4.0
		White	309	96.0
	Education	No qualifications or GCSE/CSE/O‐level	125	38.8
		A‐level or equivalent (NVQ3)	71	22.0
		Higher education (Bachelor degree, Masters, PhD)	126	39.1
	Indices of multiple deprivation decile (*n *= 253)	1 (Most deprived)	16	6.3
		2	18	7.1
		3	23	9.1
		4	27	10.7
		5	35	13.8
		6	25	9.9
		7	24	9.5
		8	27	10.7
		9	35	13.8
		10 (Least deprived)	23	9.1
Health and health‐related behaviours	History of cancer	Yes	47	14.6
		No	275	85.4
	Smoking status	Never smoked	193	59.9
		Current or ex‐smoker	129	40.1
	Fruit/vegetable consumption in past month	Less than 5 portions per day	215	66.8
		5 or more portions per day	107	33.2
	Average number of days doing 30 min of exercise in the last week		Mean = 3.1 (*SD* = 2.462)	
Screening history	Timing of last mammography	5 or more years ago	11	3.4
		3–4 years ago	40	12.4
		Within the last 2 years	271	84.2
	Bowel cancer screening in the last 2 years	Yes	254	78.9
		No	68	21.1
	Cervical cancer screening in the last 5 years	Yes	217	67.4
		No	105	32.6

*Note*: *n* = 322 unless stated otherwise.

### 3.1 Willingness to receive advice and prospective acceptability of receiving advice

Over 85% of women indicated that they would be willing to receive information about cervical cancer screening, bowel cancer screening and early symptoms of other cancers at a breast cancer screening appointment (see Table 2). Cochran's *Q* indicated that willingness to receive advice at a breast cancer screening appointment differed according to the content of that advice (*Q* = 15.2, *p*< .001). While the proportion of women willing to receive advice about bowel cancer screening (90%) and early symptoms of cancer (92%) was similar, willingness to receive advice about cervical cancer screening was significantly lower (86%, McNemar *χ* ^2^ = 11.12, *p*< .01).

**Table 2:** Willingness to receive advice

Willingness to receive advice about…		*N*	%	95% CI	Dichotomized outcome	%	95% CI
Cervical cancer screening	No, definitely not	13	4.0	2.2–6.5	Not willing	14.0	10.6–18.0
	No, probably not	15	4.7	2.5–7.1			
	Not sure	17	5.3	2.8–7.8			
	Yes, probably	95	29.5	24.5–34.5	Willing	86.0	82.0–89.4
	Yes, definitely	182	56.5	51.2–62.1			
Bowel cancer screening	No, definitely not	8	2.5	0.9–4.3	Not willing	9.9	6.8–13.0
	No, probably not	8	2.5	0.9–4.3			
	Not sure	16	5.0	2.8–7.5			
	Yes, probably	103	32.0	27.0–37.3	Willing	90.1	87.0–93.2
	Yes, definitely	187	58.1	52.8–63.4			
Early symptoms of cancer	No, definitely not	3	0.9	0.0–2.2	Not willing	8.4	5.3–11.5
	No, probably not	9	2.8	0.9–4.7			
	Not sure	15	4.7	2.5–7.1			
	Yes, probably	100	31.1	26.1–35.7	Willing	91.6	88.5–94.7
	Yes, definitely	195	60.6	55.3–65.8			

Abbreviation: CI, confidence interval.

**Figure 1:**
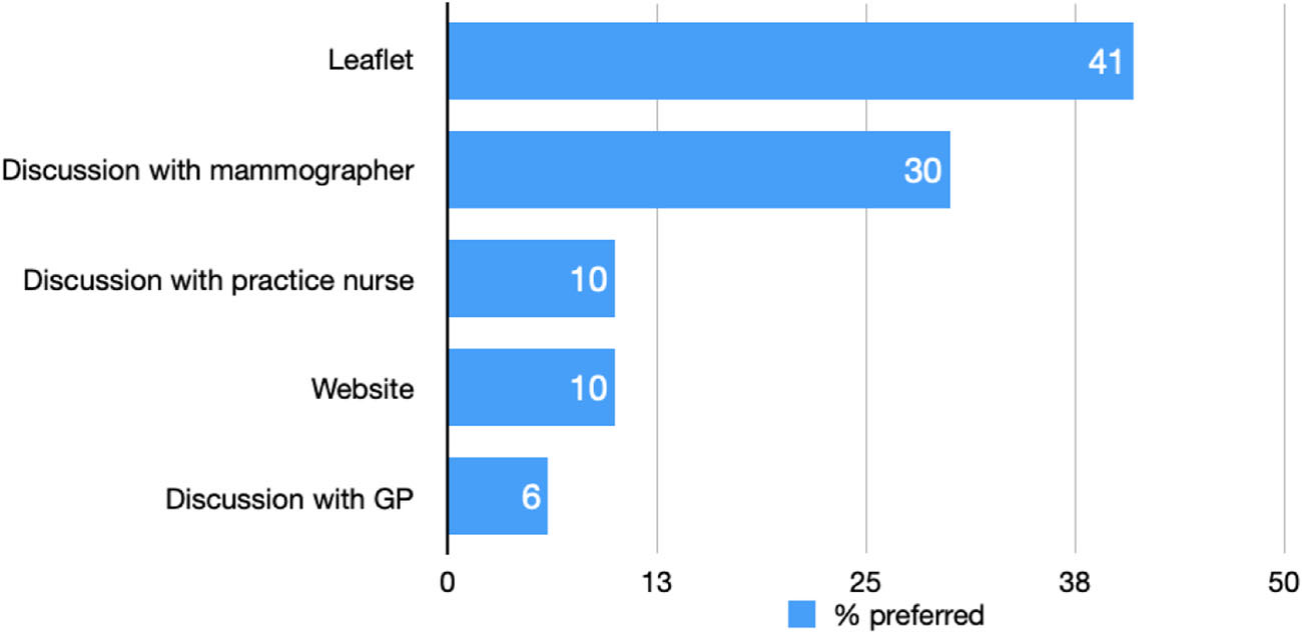
Preferred mode of advice

**Figure 2:**
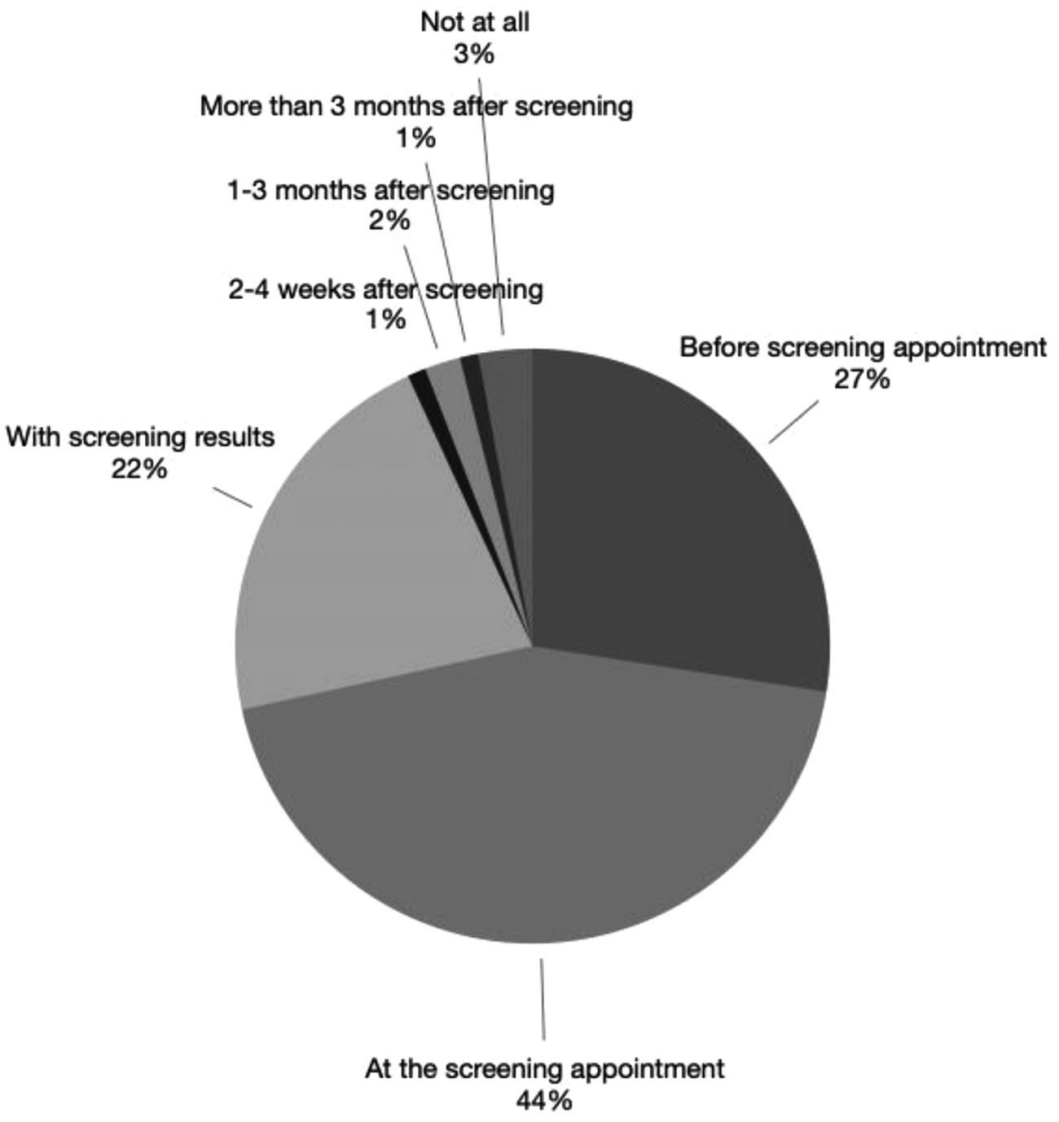
Preferred timing of advice

The preferred ways to receive advice were by leaflet (preferred by 41% of women) and discussion with the mammographer (preferred by 30% of women) and before the appointment (preferred by 27% of women), at the appointment (preferred by 44% of women) or with results (preferred by 22% of women; see Figures 1 and 2).

Cognitive and affective acceptability were high for all three forms of advice (see File S3). The Wilcoxon signed ranks test indicated that cognitive acceptability of receiving advice about early symptoms of cancer was higher than that for receiving advice about bowel cancer screening (*z* = −6.89, *p*< .001), which in turn was higher than that for receiving advice about cervical cancer screening (*z* = −5.12, *p*< .001). Affective acceptability was similar across the three types of advice.

### 3.2 Potential for adverse impact of receiving advice on breast cancer screening participation

Thirteen percent (95% confidence interval [CI]: 9.3–16.8) of women indicated that the prospect of receiving advice about cervical cancer may reduce their likelihood of attending a breast screening appointment. A similar proportion (10.9%, 95% CI: 7.5–14.3, Cochran's *Q *= 5.8, *df* = 2; *p*= .056) indicated that the prospect of receiving advice about bowel cancer screening or early symptoms of other cancers could reduce the likelihood of participation in the breast cancer screening programme.

### 3.3 Factors associated with willingness to receive advice

Univariate logistic regression (see Table 3) indicated that those who were *not* up to date with breast screening and cervical cancer screening were less willing to receive advice about cervical cancer screening at a breast cancer screening appointment. Higher cognitive and affective acceptability were associated with increased willingness to receive such advice. Multivariable logistic regression indicated that not having attended cervical screening in the past 5 years (odds ratio [OR] = 0.34, 95% CI: 0.15–0.77; *p*< .05) and cognitive acceptability (OR = 1.32, 95% CI: 1.20–1.45; *p*< .001) were independent factors associated with willingness to receive advice about cervical cancer screening.

**Table 3:** Factors associated with willingness to receive information (univariate analyses)

Variable	Willing to receive information about…														
	Cervical cancer screening					Bowel cancer screening					Early symptoms of cancer				
	*B*	*SE*	Odds ratio	95% CI	*p* Value	*B*	*SE*	Odds ratio	95% CI	*p* Value	*B*	*SE*	Odds ratio	95% CI	*p* Value
Age	0.01	0.115	1.01	0.81–1.27	.903	0.12	0.133	1.02	0.78–1.32	.906	−0.05	0.145	0.95	0.71–1.26	.711
Marital status															
Married or civil partner	Ref.	–	–	–	–	Ref.	–	–	–	–	Ref.	–	–	–	–
Separated or divorced	0.24	0.484	1.02	0.40–2.64	.961	−0.63	0.480	0.53	0.21–1.37	.192	−0.45	0.504	0.64	0.24–1.71	.372
Single	−0.68	0.432	0.51	0.22–1.18	.117	−0.66	0.509	0.52	0.19–1.41	.198	0.55	0.768	1.74	0.39–7.82	.473
Widowed	−0.34	0.586	0.71	0.23–2.26	.560	−0.02	0.781	0.98	0.21–4.52	.978	0.02	0.781	0.98	0.21–4.52	.978
Ethnicity															
White	Ref.	–	–	–	–	Ref.	–	–	–	–	Ref.	–	–	–	–
All other ethnic groups combined	0.69	1.053	1.99	0.25–15.71	.513	−0.53	0.792	0.59	0.13–2.80	.507	−0.73	0.797	0.48	0.10–2.31	.362
Education															
No qualifications/GCSE/CSE/O‐level	Ref.	–	–	–	–	Ref.	–	–	–	–	Ref.	–	–	–	–
A‐level or equivalent	−0.67	0.412	0.51	0.23–1.15	.105	0.06	0.494	1.06	0.40–2.80	.904	0.04	0.531	1.05	0.37–2.96	.933
Higher education	−2.12	0.385	0.81	0.38–1.72	.582	0.10	0.422	1.10	0.48–2.52	.817	0.11	0.456	1.12	0.46–2.74	.805
Indices of deprivation (*n* = 253)															
Most deprived (deciles 1–5)	Ref.	–	–	–	–	Ref.	–	–	–	–	Ref.	–	–	–	–
Least deprived (deciles 6–10)	0.35	0.375	1.41	0.68–2.95	.356	0.22	0.422	1.25	0.55–2.85	.601	0.01	0.478	1.02	0.40–2.59	.976
Timing of last mammography															
Within the last 2 years	Ref.	–	–	–	–	Ref.	–	–	–	–	Ref.	–	–	–	–
3‐4 years ago	−0.43	0.456	0.65	0.27–1.60	.351	−0.39	0.524	0.68	0.24–1.90	.462	−0.85	0.503	0.43	0.16–1.14	.091
5 or more years ago	**−1.79**	**0.633**	**0.17**	**0.05–0.58**	**<.01**	−1.35	0.710	0.26	0.06–1.04	.057	−1.08	0.817	0.34	0.07–1.68	.186
Bowel screening (last 2 years)															
Yes	Ref.	–	–	–	–	Ref.	–	–	–	–	Ref.	–	–	–	–
No	−0.22	0.377	0.80	0.38–1.68	.556	**−1.52**	**0.385**	**0.22**	**0.10–0.47**	**<.001**	**−0.88**	**0.425**	**0.42**	**0.18–0.96**	**<.05**
Cervical screening (last 5 years)															
Yes	Ref.	–	–	–	–	Ref.	–	–	–	–	Ref.	–	–	–	–
No	**−1.45**	**0.335**	**0.23**	**0.12–0.45**	**<.001**	−0.53	0.378	0.59	0.28–1.23	.160	**−1.21**	**0.412**	**0.30**	**0.13–0.66**	**<.01**
History of cancer															
No	Ref.	–	–	–	–	Ref.	–	–	–	–	Ref.	–	–	–	–
Yes	0.12	0.470	1.13	0.45–2.84	.796	1.01	0.748	2.76	0.64–11.94	.176	0.81	0.752	2.25	0.52–9.83	.281
Cognitive acceptability	**0.32**	**0.045**	**1.37**	**1.26–1.50**	**<.001**	**0.30**	**0.047**	**1.34**	**1.23–1.47**	**<.001**	**0.28**	**0.048**	**1.32**	**1.20–1.45**	**<.001**
Affective acceptability	**0.25**	**0.041**	**1.29**	**1.19–1.39**	**<.001**	**0.32**	**0.051**	**1.38**	**1.25–1.53**	**<.001**	**0.31**	**0.054**	**1.37**	**1.23–1.52**	**<.001**

*Note:* Bold indicates a significance of *p* < .05.

Abbreviation: CI, confidence interval.

With regard to advice about bowel cancer screening, in the univariate analysis, women who were not up to date with bowel screening were less willing to receive advice about bowel cancer screening, although this was not significant at the multivariable level. Higher cognitive and affective acceptability were associated with increased willingness to receive advice about bowel cancer screening, and these remained independent factors in the multivariable analysis (cognitive acceptability: OR = 1.27, 95% CI: 1.15–1.39; *p*< .001; affective acceptability: OR = 1.23, 95% CI: 1.09–1.38; *p*< .01).

With regard to willingness to receive information about symptoms of other cancers, women were less willing to receive this advice if they were not up to date with bowel and cervical cancer screening. Again, higher cognitive and affective acceptability were associated with increased willingness to receive advice. No cervical cancer screening within the last 5 years (OR = 0.34, 95% CI: 0.13–0.88; *p*< .05), and cognitive (OR = 1.25; 95% CI: 1.11–1.40; *p*< .001) and affective acceptability (OR = 1.16, 95% CI: 1.02–1.32; *p*< .05) remained significantly associated at the multivariable level.

## 4 DISCUSSION

In this survey of women eligible for all three UK national cancer screening programmes, there were high levels of willingness and acceptability towards receiving advice about screening and early detection of other cancers alongside breast cancer screening. Women would prefer such advice to be provided through a leaflet or discussion with the mammographer at the breast cancer screening appointment although some would prefer the information to be provided before the appointment or with their breast screening results. Existing interventions that have utilized the breast cancer screening programme as a teachable moment have focused on early detection of breast cancer by raising awareness and encouraging early presentation of symptoms^21^ or prevention.^22^ The current study extends this by considering the early detection of different cancers. The high levels of willingness and acceptability are promising and comparable to previous findings that have looked into willingness to receive lifestyle advice as part of screening programmes.^17^, ^18^ However, there remains a proportion of women who would not be willing to receive advice and, more concerningly, approximately 13% may be reluctant to attend their breast screening appointment if they knew that advice about screening and early detection of other cancers would be provided. This concurs with the findings about the potential to give lifestyle advice as part of the breast or cervical screening programmes where a minority of people reported that provision of advice would make them less willing to participate in future cancer screening.^17^ Any benefits of such an intervention in terms of uptake of other screening or prompt help‐seeking would thus need to be carefully weighed up against the potential risk of reduced breast screening uptake.

The new measures of cognitive and affective acceptability underpinned by the TFA^16^ were successful in determining similarities and differences in acceptability between different types of advice and the measure showed construct validity in the associations with willingness to receive advice. There is scope to test the measures further to determine predictive validity and whether statistically significant differences in affective or cognitive acceptability translate into meaningful real‐world differences. Prospective acceptability was a key factor associated with willingness to receive advice, offering further support for the TFA and reinforcing the importance of ensuring that interventions are acceptable to the target group. Further work could focus on the practitioner's views of acceptability and feasibility, or adapt the measures to assess concurrent and retrospective cognitive and affective acceptability of patients.

Women were less willing to receive advice about cervical cancer screening compared to advice about bowel cancer screening or symptoms of other cancers, and the prospect of receiving advice about cervical cancer screening was found to be the least acceptable. This suggests that there are certain issues with cervical screening advice that may not be present in advice about bowel screening and early symptoms of different types of cancer. This could be because women in this age group see cervical screening as less necessary or it could be a result of a range of previously documented concerns about cervical screening,^23^ whereas a key concern for bowel screening is embarrassment over providing a stool sample.^19^, ^24^ Cervical cancer screening is more invasive, and there are also specific barriers to cervical cancer screening in this age group such as increased discomfort and low perceived risk.^25^, ^26^ Self‐sampling approaches may overcome some of these issues, and women's willingness to be offered a self‐sampling kit for HPV testing at the mammography appointment could be an area for future research. It is notable that affective acceptability was similar across the different types of advice, but cognitive acceptability was lower. This suggests that there is something about perceived burden, coherence, benefits, costs and efficacy of the intervention that differs rather than the potential for anxiety, judgement or embarrassment. The qualitative enquiry may offer more insight into these explanations.

In contrast to previous research that has highlighted that education and ethnicity may affect willingness to receive advice about lifestyle,^17^, ^29^ the current study found that willingness to receive advice about screening and early detection of other cancers was similar across sociodemographic groups. Health‐related behaviours were also largely unrelated to willingness to receive advice, with the exception of previous screening participation. Whether or not the women were up to date with cancer screening was a consistent factor associated with willingness to receive advice. Those who were up to date with cervical cancer screenings were more willing to receive advice about encouraging cervical cancer screening and advice about early symptoms of other cancers. This is key as it may mean that those most in need of the advice are those least willing to receive it, reminiscent of the inverse care law.^27^ This differs from findings about interest in receiving lifestyle advice as part of the screening programme: Those whose health behaviour fell short of recommendations about weight, physical activity, smoking or alcohol consumption were found to be more interested in receiving advice.^29^

### 4.1 Strengths and limitations

These findings extend the existing literature, providing a better understanding about whether or not women find it acceptable to receive advice about screening and early detection of other cancers while at a breast cancer screening appointment, and how that in turn may affect their willingness to receive the advice. Use of the TFA to examine acceptability was helpful in identifying women's beliefs about the provision of advice. The findings from this study can help to understand the use of breast cancer screening as a teachable moment.

However, this study has limitations: The study used a self‐reported questionnaire in a cross‐sectional design; therefore, causation cannot be established. Women were not asked about hysterectomy: A proportion of respondents may have been ineligible for cervical screening, which in turn may have affected recent screening attendance and willingness to receive advice about cervical cancer screening. The data were also collected in June 2020 when the national breast cancer screening programme had been paused in the United Kingdom due to the COVID‐19 pandemic. It is unknown how this may have affected the data. Despite this, relationships could be deduced, and the sample size provided power to detect associations. A further limitation is that the sample included very few women from ethnic backgrounds other than White. It is therefore not possible to generalize the findings to the wider population, especially as women from ethnic minority backgrounds tend to show lower screening uptake.^28^ Finally, surveys often find that self‐reported screening uptake is higher than uptake reported in national data, likely due to response bias and indeed that was the case in this study. However, as previous participation in breast screening was an inclusion criterion for the study, bowel screening participation would be expected to be higher in our sample than the general population, given that women who have taken part in breast screening are more likely to participate in the other screening programmes.^30^

## 5 CONCLUSION

On the one hand, there is high willingness and high acceptability towards using breast cancer screening as a teachable moment. However, the few women who find it unacceptable and are less willing to receive the advice about screening and early diagnosis may be the intended target of such an intervention and the very women who would benefit the most.

## CONFLICT OF INTERESTS

The authors declare that there are no conflicts of interest.

## Supporting information

Supporting information.

## Data Availability

The data that support the findings of this study are available on request from the corresponding author. The data are not publicly available due to privacy or ethical restrictions.
