# Supplementary material for: “Whilst you are here…” Acceptability of providing advice about screening and early detection of other cancers as part of the breast cancer screening programme
Source: Health Expect. 2021 Aug 8;24(5):1868–78. doi: 10.1111/hex.13330 (PMC8483189; doi:10.1111/hex.13330)
Supplement: Supplementary file 1 — Supporting information. [file HEX-24-1868-s001.docx]

**SUPPLEMENTARY FILE 1**

**Items in online questionnaire (adapted from Stevens et al (2019)^18^)**

| **Question** | **Response options** |
| --- | --- |
| What is your exact age? |  |
| What is your postcode? |  |
| What is your marital status? | Single Married or civil partner  Separated or divorced Widowed |
| What is your ethnic group? | White: English/Welsh/Scottish/Northern Irish/British White: Irish White: Gypsy or Irish Traveller White: Any other White background   Multiple ethnic group: White and Black Caribbean Multiple ethnic group: White and Black African Multiple ethnic group: White and Asian Multiple ethnic group: Any other Mixed/Multiple ethnic background   Asia / Asian British: Indian  Asia / Asian British: Pakistani  Asia / Asian British: Bangladeshi Asia / Asian British: Chinese  Asia / Any other Asian background  African/Caribbean/Black British: African African/Caribbean/Black British: Caribbean African/Caribbean/Black British: Any other Black/Caribbean background   Other ethnic group: Arab  Other: |
| What is the highest level of educational qualification you have obtained? | No qualifications GCSE/O Level/CSE Vocational Qualifications (NVQ1 + 2) A Level or equivalent (NVQ3) Bachelor degree or equivalent (NVQ4) Masters/PhD or equivalent Other: |
| Over the past month, how many portions of fruit or vegetables did you usually eat each day? Please include fruit eaten at meal times or as a snack  *Examples of a portion are: 1 apple or banana A large slice of melon 2 plums or satsumas A small bowl of grapes 2 tablespoons of tinned fruit  ½ tablespoon of dried fruit*  *2 heaped tablespoons of broccoli or carrots*  *3 tablespoons of sweetcorn or peas*  *A bowl of salad* | 0 portions per day 1 portion per day 2 portions per day 3 portions per day 4 portions per day 5 or more portions per day |
| Do you smoke? | No, I have never smoked  No, but I used to smoke  Yes I smoke (every day or occasionally) |
| In the past week on how many days have you done a total of 30 minutes or more of physical activity, which was enough to raise your breathing rate?  *This may include sport, exercise, and brisk walking or cycling for recreation of to get to and from places, but should not include housework or physical activity that may be part of your job.* | None 1 day 2 days 3 days 4 days 5 days 6 days 7 days |
| Have you ever been to a breast cancer screening appointment?   *Breast cancer screening (mammography) involves checking for breast cancer in women who have no signs or symptoms of the disease. In the UK you are invited every 3 years once you become 50 years old* | Yes No |
| When was your most recent breast cancer screening? | Less than a year ago 1-2 years ago 3-4 years ago 5 or more years ago Not applicable. I have never been screened for breast cancer. |
| Have you ever been screened for bowel cancer in the last 2 years?  *There are two types of tests used in NHS bowel cancer screening: (1) a home testing kit where you provide a sample of your poo. (2) bowel scope screening which involves a thin flexible tube with a camera at the end that looks for and removes any polyps inside your bowel* | Yes, a home testing kit Yes, a bowel scope screening No  Do not know |
| Have you ever been screened for cervical cancer in the past 5 years?  *At a cervical screening appointment, a nurse takes a sample of cells from your cervix using a small, soft brush (it’s also called a Pap smear test).* | Yes No Do not know |
| Have you ever been diagnosed with cancer? | Yes No Prefer not to say |
| **These questions are about what you think about receiving advice about early detection of other cancers when you are at a breast cancer screening (mammography) appointment.** | |
| Would you be willing to receive advice about cervical cancer screening when you are at a breast screening appointment? | No, definitely not  No, probably not  Not sure  Yes, probably  Yes, definitely |
| Would you be willing to receive advice about bowel cancer screening when are you at a breast screening appointment? |  |
| Would you be willing to receive advice about early symptoms of different types of cancer when you are at a breast screening appointment? |  |
| If you knew you would receive advice about cervical cancer screening as part of a breast screening appointment, would this make you less likely to attend breast screening? |  |
| If you knew you would receive advice about bowel cancer screening as part of a breast screening appointment, would this make you less likely to attend breast screening? |  |
| If you knew you would receive advice about early symptoms of different types of cancer as part of a breast screening appointment, would this make you less likely to attend breast screening? |  |
| Receiving advice about **[CERVICAL CANCER SCREENING / BOWEL CANCER SCREENING / EARLY SYMPTOMS OF DIFFERENT TYPES OF CANCER]** when at a breast screening appointment… | |
| …Would make me feel anxious *[REVERSE SCORED]* | Strongly disagree  Disagree  Neither agree or disagree  Agree  Strongly agree |
| …Would make me feel judged *[REVERSE SCORED]* |  |
| …Would make me fearful *[REVERSE SCORED]* |  |
| …Would make me feel embarrassed *[REVERSE SCORED]* |  |
| …Would take too much time *[REVERSE SCORED]* |  |
| …Would reassure me |  |
| …Has benefits that outweigh the costs |  |
| …Is not a priority for me *[REVERSE SCORED]* |  |
| …Would be stressful for me *[REVERSE SCORED]* |  |
| …Is important |  |
| …Is a good idea |  |
| …Would [**make me** **more likely to take part in cervical cancer screening /make me more likely to take part in bowel cancer screening / help me know when to consult the doctor with symptoms**] |  |
| …Makes sense to me |  |
| When would you prefer to receive advice on early detection of other cancers as part of the breast cancer screening programme? | Before I attend the breast screening appointment  At the breast screening appointment  With my breast screening results (around two weeks after attending screening)  2-4 weeks after attending breast screening  1-3 months after attending breast screening  More than 3 months after attending breast screening  Not at all |
| How would you prefer receive advice on early detection of other cancers as part of the breast cancer screening programme? | Video  Leaflet  Website  App  Discussion with mammographer (practitioner who does the breast screening)  Discussion with my GP  Discussion with my practice nurse |

**SUPPLEMENTARY FILE 2: Development and psychometric testing of acceptability measure**

***Exploratory principal components analysis***

The 13 items concerning acceptability of receiving advice about bowel cancer screening were entered into the exploratory principal components analysis (varimax rotation with Kaiser normalisation) to determine the structure of the data. The overall Kaiser-Meyer-Olkin (KMO) and Bartlett’s test of sphericity indicated the items shared a common factor and the data was suitable for principal components analysis (KMO = 0.901; Bartlett’s test of sphericity, Chi sq= 3386.33, df=78, p<0.001). The anti-image correlations matrix indicated that the item specific KMO statistics were all greater than 0.5 demonstrating satisfactory sampling adequacy. Using Eigenvalues greater than 1.0 as a basis for factor selection, the 13 items loaded onto 2 factors and all factor loadings were greater than 0.5 (see **Table 1**). The 2 factors explained 69.8% of the total variance in the 13 items. Eight items converged onto one factor - this was assigned the label of ‘cognitive acceptability’. The remaining five items converged on to a second factor which was assigned the label of ‘affective acceptability’.

**Table 1. Rotated component matrix after exploratory principal components analysis**

| Item | Component (factor) | |
| --- | --- | --- |
|  | 1 | 2 |
| **Factor 1: Cognitive acceptability** |  |  |
| …would reassure me | **0.716** | 0.287 |
| …has benefits that outweigh the costs | **0.780** | 0.247 |
| …is important | **0.849** | 0.196 |
| …is a good idea | **0.867** | 0.217 |
| …would make me more likely to take part in bowel cancer screening | **0.723** | 0.089 |
| …makes sense to me | **0.848** | 0.282 |
| …is not a priority for me | **0.551** | 0.401 |
| …would take too much time | **0.569** | 0.416 |
| **Factor 2: Affective acceptability** |  |  |
| …would make me feel anxious | 0.228 | **0.873** |
| …would make me feel judged | 0.360 | **0.816** |
| …would make me feel fearful | 0.182 | **0.911** |
| …would make me feel embarrassed | 0.236 | **0.812** |
| …would be stressful for me | 0.241 | **0.848** |

***Confirmatory principal components analysis***

For the confirmatory principal components analysis, responses to the 13 items concerning acceptability of receiving advice about early symptoms of cancer were used.

The overall Kaiser-Meyer-Olkin (KMO) and Bartlett’s test of sphericity indicated the items shared a common factor and the data was suitable for principal components analysis (KMO = 0.911; Bartlett’s test of sphericity, Chi sq= 3682.17, df=78, p<0.001).

Extraction was based on a specified number of factors (2) and we hypothesised that the 8 cognitive acceptability items would form one factor and the 5 affective acceptability items would load onto a second factor. Factor loadings confirmed this structure (see **Table 2**) and all factor loadings were greater than 0.6.

Confirmatory principal components analysis was repeated for the 13 items concerning acceptability of receiving advice about cervical cancer screening. Factor loadings again confirmed the hypothesised structure and all factor loadings were greater than 0.7 with the exception of *“…would take too much time”* (0.534) and *“…is not a priority for me”* (0.582).

**Table 2. Rotated component matrix after confirmatory principal components analysis**

| Item | Component (factor) | |
| --- | --- | --- |
|  | 1 | 2 |
| **Factor 1: Cognitive acceptability** |  |  |
| …would reassure me | **0.730** | 0.311 |
| …has benefits that outweigh the costs | **0.846** | 0.235 |
| …is important | **0.852** | 0.216 |
| …is a good idea | **0.845** | 0.287 |
| …would help me to know when to consult the doctor with symptoms | **0.766** | 0.276 |
| …makes sense to me | **0.818** | 0.337 |
| …is not a priority for me | **0.605** | 0.246 |
| …would take too much time | **0.626** | 0.354 |
| **Factor 2: Affective acceptability** |  |  |
| …would make me feel anxious | 0.235 | **0.895** |
| …would make me feel judged | 0.428 | **0.714** |
| …would make me feel fearful | 0.224 | **0.907** |
| …would make me feel embarrassed | 0.383 | **0.704** |
| …would be stressful for me | 0.292 | **0.813** |

***Description and internal reliability of the two factors***

*Cognitive acceptability*

Eight items reflect elements of the perceived burden, coherence, opportunity costs and efficacy of the intervention. Higher scores on this sub-scale (range 8-40) indicate a higher level of acceptability. This subscale demonstrated good internal reliability in relation to receiving advice about cervical cancer screening (Cronbach’s alpha = 0.88), bowel cancer screening (Cronbach’s alpha = 0.90) and symptoms of cancer (Cronbach’s alpha = 0.92).

*Affective acceptability*

Five reverse-scored items reflect the perceived emotional consequence of the intervention. Higher scores on this sub-scale (range 5-25) indicate lower level of negtaive emotions and as such a higher level of acceptability. This subscale demonstrated good internal reliability in relation to receiving advice about cervical cancer screening (Cronbach’s alpha = 0.92), bowel cancer screening (Cronbach’s alpha = 0.93) and symptoms of cancer (Cronbach’s alpha = 0.92).

**SUPPLEMENTARY FILE 3**

| **Cognitive Acceptability**  **(possible range: 8-40)** | 31.7 | 5.71 | 32.0 | 32.6 | 5.8 | 32.0 | 33.6 | 5.29 | 33.0 | 106.3 | <0.01 |
| --- | --- | --- | --- | --- | --- | --- | --- | --- | --- | --- | --- |
| **Affective Acceptability**  **(possible range: 5-25)** | 20.9 | 4.13 | 21.0 | 21.1 | 4.14 | 21.0 | 20.9 | 4.04 | 21.0 | 1.8 | 0.413 |

**Prospective cognitive and affective acceptability of receiving advice**
